# The Effects of Angiotensin II and Angiotensin-(1–7) in the Rostral Ventrolateral Medulla of Rats on Stress-Induced Hypertension

**DOI:** 10.1371/journal.pone.0070976

**Published:** 2013-08-14

**Authors:** Dongshu Du, Jun Chen, Min Liu, Minxia Zhu, Haojia Jing, Jie Fang, Linlin Shen, Danian Zhu, Jerry Yu, Jin Wang

**Affiliations:** 1 Department of Physiology and Pathophysiology, Shanghai Medical College, Fudan University, Shanghai, China; 2 Department of Neurobiology, School of Life Sciences, Shanghai University, Shanghai, China; 3 Department of Pathology, Changzheng Hospital, Second Military Medical University, Shanghai, China; 4 Department of Pulmonary Medicine, University of Louisville, Louisville, Kentucky, United States of America; Kaohsiung Chang Gung Memorial Hospital, Taiwan

## Abstract

We have shown that angiotensin II (Ang II) and angiotensin-(1–7) [Ang-(1–7)] increased arterial blood pressure (BP) via glutamate release when microinjected into the rostral ventrolateral medulla (RVLM) in normotensive rats (control). In the present study, we tested the hypothesis that Ang II and Ang-(1–7) in the RVLM are differentially activated in stress-induced hypertension (SIH) by comparing the effects of microinjection of Ang II, Ang-(1–7), and their receptor antagonists on BP and amino acid release in SIH and control rats. We found that Ang II had greater pressor effect, and more excitatory (glutamate) and less inhibitory (taurine and γ-aminobutyric acid) amino acid release in SIH than in control animals. Losartan, a selective AT_1_ receptor (AT_1_R) antagonist, decreased mean BP in SIH but not in control rats. PD123319, a selective AT_2_ receptor (AT_2_R) antagonist, increased mean BP in control but not in SIH rats. However, Ang-(1–7) and its selective Mas receptor antagonist Ang779 evoked similar effects on BP and amino acid release in both SIH and control rats. Furthermore, we found that in the RVLM, AT_1_R, ACE protein expression (western blot) and ACE mRNA (real-time PCR) were significantly higher, whereas AT_2_R protein, ACE2 mRNA and protein expression were significantly lower in SIH than in control rats. Mas receptor expression was similar in the two groups. The results support our hypothesis and demonstrate that upregulation of Ang II by AT_1_R, not Ang-(1–7), system in the RVLM causes hypertension in SIH rats by increasing excitatory and suppressing inhibitory amino acid release.

## Introduction

A moden life style with fast pace can cause emotional and psychological stress, which may lead to a gradual increase in hypertension in young adults. There is compelling evidence that the renin-angiotensin system (RAS) in the brain plays a vital role in regulation of arterial blood pressure (BP) and sympathetic nerve activity. Therefore, development of strategies to regulate RAS activity in the pathophysiology of hypertension is of great interest [1]. The protease renin catalyzes angiotensinogen into an inactive decameric peptide, angiotensin I (Ang I), which is then converted to active octapeptide angiotensin II (Ang II) by angiotensin-converting enzyme (ACE). Ang II is one of the major players in the RAS, acting on various receptors such as Ang II type 1 (AT_1_R) and type 2 receptors (AT_2_R). Most effects of Ang II are mediated by AT_1_R. By binding to AT_1_R, Ang II desensitizates baroreflex and increases sympathetic outflow, BP and vasopressin release [2]. In contrast, the role of AT_2_R in central regulation of BP is not clear. Angiotensin-converting enzyme homolog (ACE2) is a new component of the RAS [3], [4]. ACE2 cleaves Ang I to Ang-(1–9) [4], which can be further converted into the heptapeptide Ang-(1–7) by ACE. ACE2 also hydrolyzes Ang II into Ang-(1–7) with a higher efficiency [∼400-fold higher than that for Ang I to Ang-(1–9)] [5]. Ang-(1–7) acts on Mas receptor, which is different from AT_1_R and AT_2_R [6]. Central Ang-(1–7) increases baroreflex sensitivity and decreases norepinephrine release and BP in hypertensive rats [7]. Many components of the RAS, including renin, ACE, ACE2, Ang II, Ang-(1–7) and their receptors are present in cardiovascular centers [8], [9]. The expression of renin, ACE, Ang II and AT_1_R were upregulated in the brain of hypertension model [10]. Endogenous Ang II and Ang-(1–7) in the rostral ventrolateral medulla (RVLM) are thought to regulate the cardiovascular activities [11]. The RVLM contains sympathetic vasomotor neurons and is a cardiovascular integration center, which receives excitatory as well as inhibitory inputs from central and peripheral sites [12]. The RVLM neurons send monosynaptic projections to the intermediolateral cell column (IML) of the spinal cord to regulate the functions of the heart, arterioles and kidneys via sympathetic nerves. Chemical or electrical stimulation of the RVLM increases sympathetic nerve activities and BP. Destruction of the RVLM causes hypotension.

Our previous studies indicated that Ang II and Ang-(1–7) in the RVLM increased cardiovascular activities and release of excitatory amino acids in normotensive rats [13]–[15]. Glutamatergic neurons in the RVLM are potential candidates for sympathetic vasomotor neurons that project to the spinal cord [13], [16]. Ang II levels in the brain, myocardium and vasculature are significantly increased in acute and chronic stress rats [17], [18]. Moreover, microinjection of AT_1_R antagonists into the RVLM attenuated the sustained phase of the pressor stress response [19]. Clearly, RAS in the RVLM are activated during stress. However, little is known concerning the relative importance of Ang II and Ang-(1–7) in stress-induced hypertension (SIH). Therefore, we hypothesized that Ang II and Ang-(1–7) in the RVLM are differentially activated in SIH rats. Indeed, our data support that Ang II, not Ang-(1–7), plays an important role in SIH.

## Materials and Methods

### Ethics Statement

The current experiments conformed to the Regulations for the Administration of Affairs Concerning Experimental Animals, National Committee of Science and Technology of China and Instructive Notions with Respect to Caring for Laboratory Animals, the Ministry of Science and Technology of China, and were approved by the Ethics Committee for Experimental Research, Shanghai Medical College, Fudan University.

### General preparation

Male Wistar rats (7 to 9 weeks old) were purchased from the animal center of the Chinese Academy of Sciences. They were kept individually well-ventilated cages at a room temperature of 22±2°C on a 12 h light/dark cycle. Housing facilities and all experimental protocols were approved by the Animals Care and Use Committee, Shanghai Medical College, Fudan University and complied with the Guide for the Care and Use of Laboratory Animals (New edition, 2012).

Rats were randomly divided into normotensive (control) and SIH groups. The SIH model was established as previous reported [20], [21]. In the SIH group, rats were placed in a cage (22 cm×22 cm×28 cm) with a grid floor and received electric foot-shocks (55∼90 V, 50∼100 ms duration, 2∼30 s intervals) and/or noises (88∼98 db) produced by a buzzer randomly delivered by a computer. A 2 h electric shock session was applied twice daily with a 4 h interval for l5 consecutive days. The control rats received the same treatment excluding the stressful stimuli. Systolic blood pressure (SBP) was measured with the tail-cuffed method under conscious condition. Measurements were repeated three times to obtain an average value. In the SIH group, rats with SBP below 140 mmHg on the 15^th^ day were excluded from the experiment. At the day 15, rats were anesthetized with urethane (700 mg/kg) mixed with α-chloralose (35 mg/kg) intraperitoneally.

### Recording/measuring arterial blood pressure

The trachea was cannulated. The right carotid artery was cannulated for continuous recording of arterial blood pressure (BP) with a bioelectric signals processing system (Model SMUP-A, Department of Physiology, Shanghai Medical College, Fudan University). The heart rate (HR) was derived automatically from the wave of BP. The rectal temperature was maintained at 37°C by a thermistat.

### Microinjection and microdialysis

To perform microinjection, a stainless-steel guiding tube (internal diameter of 0.12 mm) was first placed 2 mm above the injection site. An injection cannula (outside diameter 0.1 mm) was then lowered through the guiding tube for injection. The cannula was removed, refilled, and reinserted into the same position for repeated injections. Drugs were dissolved in an artificial cerebrospinal fluid (ACSF, pH 7.4, composition in mM: NaCl 130, KCl 2.99, CaCl_2_ 0.98, MgCl_2_•6H2O 0.80, NaHCO_3_ 25, Na_2_HPO_4_•12H_2_O 0.039, NaH_2_PO_4_•2H_2_O 0.46). Each drug (0.1 μl) was given with a microinjector at a constant rate over 20 s. After each injection, the needle remained in the RVLM for 5 min to avoid possible backflow of the solution.

The trachea and esophagus above the tracheal cannula were ligated, sectioned and retracted rostrally. After retraction of the bilateral longus capitis muscles, the inferior occipital bone was removed to expose the surface of the ventral medulla oblongata. Animals were placed in the supine position with the head mounted in a stereotaxic frame (51600, Stoelting, American). The RVLM was located 0.6∼1.0 mm ahead of the most rostral rootlet of the hypoglossal nerve, 1.6∼2.0 mm lateral to the midline, and 0.5∼0.8 mm below the ventral surface. The RVLM was verified by a pressor response to L-glutamate (2 nmol/0.1 μl) microinjection, as described previously [14].

As BP and HR were monitored, microdialysis was performed to examine the effect of Ang II, Ang-(1–7) or ACSF on release of amino acid neurotransmitters in the RVLM. A microdialysis probe (membrane length 0.5 mm, outside diameter 0.2 mm, EICOM, Japan) was inserted through a stainless steel guiding tube at an Angle, such that the tips of both microinjection cannula and microdialysis probe were positioned at the same site in the RVLM. Microdialysis was performed by perfusing the probe with the ACSF at a rate of 2 μl/min with a microdialysis pump (Bioanalytical Systems, MD-1001, USA). The total volume of each dialysate sample was 20 μl. The dialysate samples were collected at 10 min before and immediately following microinjection of Ang II, Ang-(1–7) or the ACSF, as described previously [14], [15].

### High-performance liquid chromatography (HPLC)

The amino acids in the microdialysis sample were separated by HPLC (LC-6A UQUID Chromatograph, Shimadzu Corporation, Japan) with a reverse-phase column (C18, Ultrasphere ODS, 4.6 mm×25 cm, particles 5 μm), and quantified with o-phthaldialdehyde derivative and fluorescence detection (RF-10AXL Shimadzu Fluorescence detector, RFU 0.01, excitation wave-length 330 nm and emission wave-length 450 nm). The mobile phase was composed of 0.1 M of potassium phosphate 63% (pH 6.00∼6.25), methanol 35%, tetrahydrofuran 2%, and the flow rate was 1 ml/min. The experiments were performed at 19∼23°C [15].

### Histology

At the end of each experiment, the microinjection and microdialysis sites in the RVLM were marked by microinjection of 2% pontamine sky blue. Then the animal was euthanized by an overdose injection of urethane and thoracotomy. The brain was removed from the skull, fixed in 10% formalin for 4 days or frozen sectioned coronally at 40 μm, and stained with Neutral Red. Location of each site studied was identified and mapped on diagrams of the rat brain from the atlas of Paxinos and Watson (Paxinos G, Watson C, 2009).

### Western blotting

The RVLM tissue was punched according to the rat brain atlas. All tissue samples were then stored at −80°C for Western blot analysis. A total protein extract was prepared by homogenizing RVLM tissue in lysis buffer with protease inhibitor. The concentration of total protein was determined by the bicinchoninic acid assay. Protein samples (40 μg) were separated by 10% sodium dodecyl sulfate-polyacrylamide gel electrophoresis (SDS-PAGE), transferred to polyvinylidene difluoride membranes (Gelman-Pall, Ann Arbor, MI, USA), and then blocked with 5% nonfat milk. The poly-vinylidene fluoride membranes (Millipore, MA, USA) were incubated with primary antibody overnight at 4°C. Primary antibodies were rabbit polyclonal antibodies against AT_1_R (1∶2000) (Alomone, Israel), rabbit polyclonal antibody against AT_2_R (1∶1000) (Santa Cruz, USA) [22], rabbit polyclonal antibodies against Mas Receptor (1∶2000) (Alomone, Israel) [23], mouse monoclonal antibody against ACE (1∶1000) (abcam, USA) [24], goat polyclonal antibody against ACE2 (1∶1000) (Santa Cruz, USA), and mouse polyclonal antibodies against β-actin (1∶3000) (as an internal standard from Beyotime Institute of Biotechnology, Haimen, China). Peroxidase-conjugated anti-rabbit, anti-mouse and anti-goat antibodies were used as the secondary antibody (1∶2000) (Millipore, MA, USA). The membranes were probed with an ECL-Plus detection kit (Beyotime Institute of Biotechnology, Haimen, China) and then scanned with Imagequant LAS4000 mini (GE Healthcare Life Sciences, CT, USA). The intensity was analyzed by a gel-pro Analyser (FURI Science and Technology, Shanghai, China).

### Real-time PCR

The total RNA was extracted from the RVLM using TRI Reagent (Invitrogen life technologies, Inc., USA). Following reverse transcription of total RNA, real-time RT-PCR was performed using the iCycler iQ Multicolor Real-Time Detection System with output to a computer-based acquisition system (Bio-Rad). The protocol consisted of denaturation (95°C for 5 min), amplification and quantification repeated 55 times (95°C for 10 s and 55°C for 45 s), denaturation at 95°C for 1 min, reannealing at 60°C for 1 min. For ACE, the sense primer was 5′-CAGGGATGGACACCCAGAAG-3′ and the antisense primer was 5′-CCATTT CGTGGTGGGCTATC-3′. For ACE2, the sense primer was 5′-CACCTTACGAGCCTCCTGTC-3′ and the antisense primer was 5′-CTGCGTTACTTTCTCCTTTGC-3′. For GAPDH (the reference gene) the sense primer was 5′-GGAAAGCTGTGGCGTGAT-3′ and the antisense primer was 5′-AAGGTGGAAGAATGGGAGTT-3′. The final results were expressed as the ratio of mRNA of interest to GAPDH.

### Statistical analysis

T-test was used for comparing the baseline data and the difference between pre- and post-injections, or between SIH rats and control rats. One way ANOVA followed by the Newman-Keuls test for post hoc analysis was used when multiple comparisons were made (GraphPad Prism 5). All data are expressed as means ± SEM. *P*<0.05 was considered statistically significant.

## Results

### 1. Changes in SBP, HR, protein expressions of ACE and AT_1_R in the SIH rats

**Figure 1:**
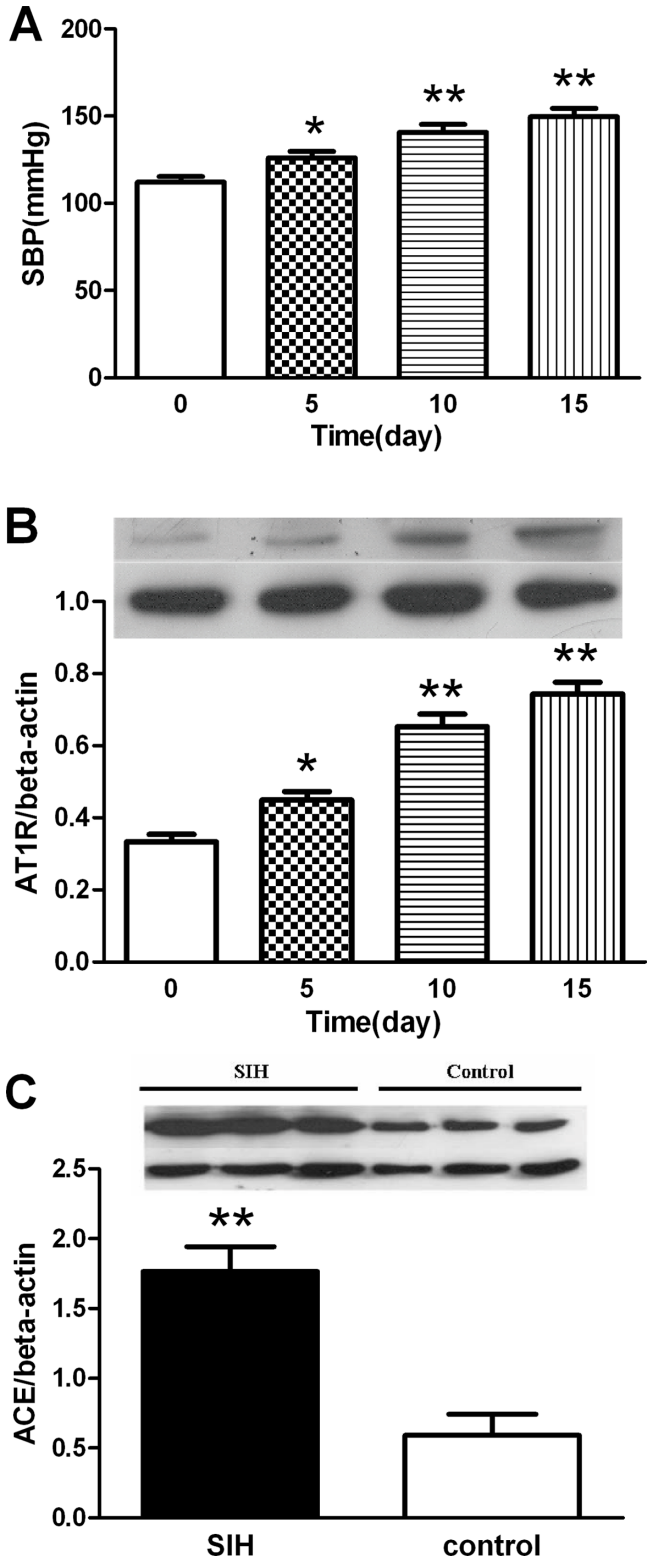
Changes in SBP and protein expressions of ACE and AT_1_R in the RVLM of stressed rats. *P<0.05, **P<0.01, compared with the control group (0 day).

We determined changes in Systolic blood pressure (SBP), heart rate (HR), protein expressions of ACE and AT_1_R in the RVLM in the SIH rats. We found that SBP and AT_1_R protein expression in the stressed rats increased in a time-dependent manner (Fig. 1A, B). Changes in SBP, HR and protein expression of AT_1_R became significant on the 5^th^ day (*P<*0.05) (Fig. 1A, B, Table 1). ACE protein expression in the RVLM increased on the 15^th^ day (Fig. 1C).

**Table 1:** Effects of microinjection of Ang II and Ang-(1–7) into the RVLM on HR.

Time	ACSF	Ang II	SIH+ Ang II	Ang- (1–7)	SIH+ Ang-(1–7)
baseline	365±12	372±23	436±20* ^#^	364±15	441±21* ^#^
after injection	369±16	381±27	448±19* ^#^	375±23	453±24* ^#^

* *P*<0.05 compared with the artificial cerebrospinal fluid (ACSF) group; ^#^ *P*<0.05 compared with the normotensive animal (n = 8 in each group).

### 2. Effects of microinjection of RAS components

**Figure 2:**
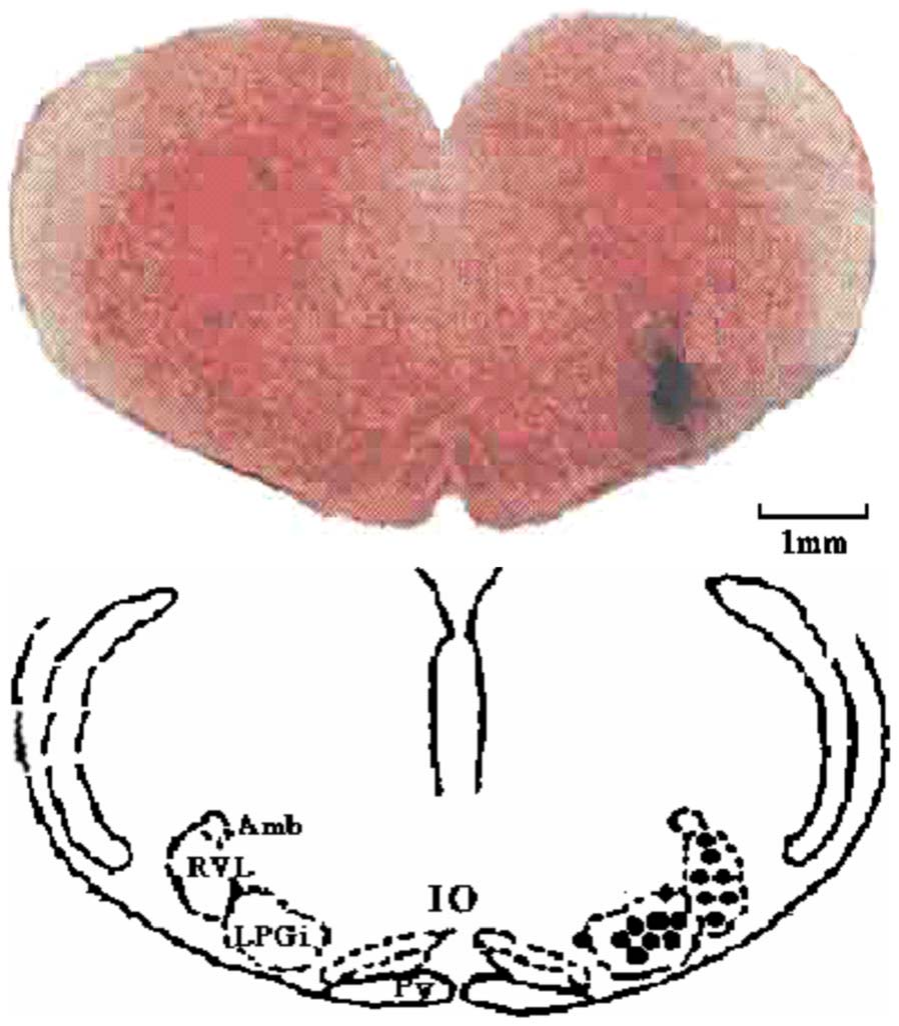
Location of microinjection and microdialysis in the RVLM. Top, a photomicrograph shows a microinjection site (black shaded area); bottom, a drawing shows coronal section (caudal to the bregma by 11.96 mm) (Paxinos and Watson atlas). Each solid point represents a site of microinjection or microdialysis in the RVLM. Amb, ambiguous nucleus; RVL, rostroventrolateral nucleus; LPGi, lateral paragigantocellular nucleus; Py, pyramidal tract; IO, inferior olive.

To determine the relative effects of Ang II and Ang-(1–7) in hemodynamic homeostasis, we examined effects of microinjection of Ang II or Ang-(1–7), and their antagonists into the RVLM on mean arterial blood pressure (MAP) and HR in SIH and control rats. Injection sites were verified (Fig. 2). Data from injections made outside of the RVLM were excluded from analysis.

**Figure 3:**
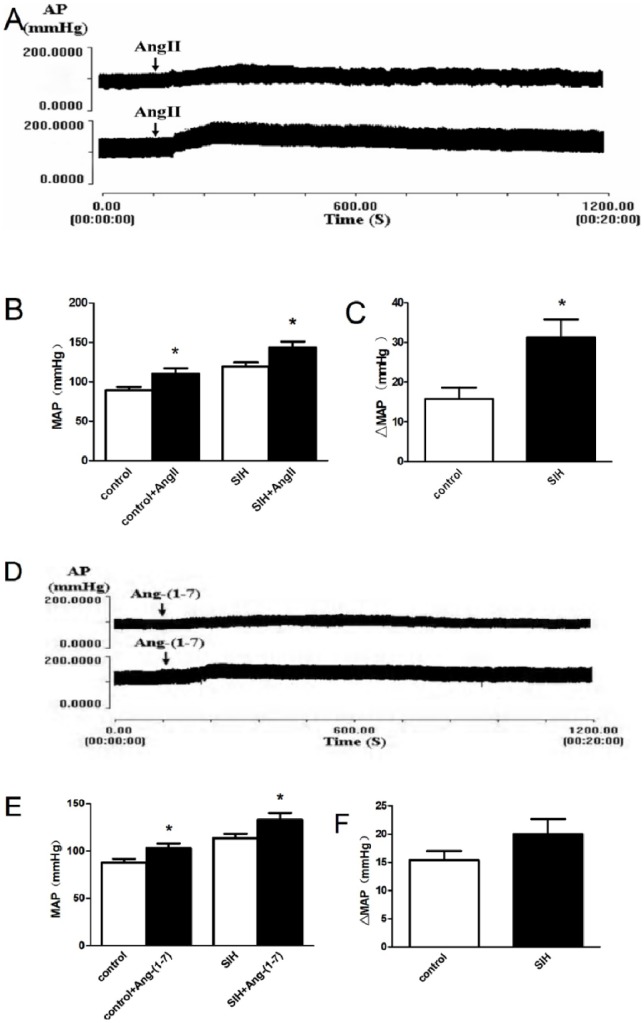
Effects of microinjection of Ang II and Ang-(1–7) into the RVLM on BP. Original **t**racings of arterial blood pressure (AP) in control (top trace in A and D) and SIH (bottom trace in A and D) rats. Arrows indicate injection time. B, C, E, and F are group data (n = 8 in each group). *P<0.05, compared with baseline (prior to microinjection) in B and E and with normotensive group in C and F, respectively.

Microinjection of Ang II (Sigma, USA, 100 pmol) into the RVLM increased MAP, which reached its peak at the time points 3∼4 minute following the microinjection, in both SIH and control rats. The BP returned to pre-injection levels after about 16 min in the control group and 26 min in the SIH group. The pressor effect was significantly greater in the SIH group than the control group (Fig. 3A, B, C) (*P*<0.05). Conversely, microinjection of Ang-(1–7) (Sigma, USA, 100 pmol) elicited similar pressor effects in both SIH and control groups (Fig. 3D, E, F). Furthermore, microinjection of Ang II or Ang-(1–7) did not significantly change HR and produced similar changes in ΔHR in SIH and control rats (Table 1).

**Figure 4:**
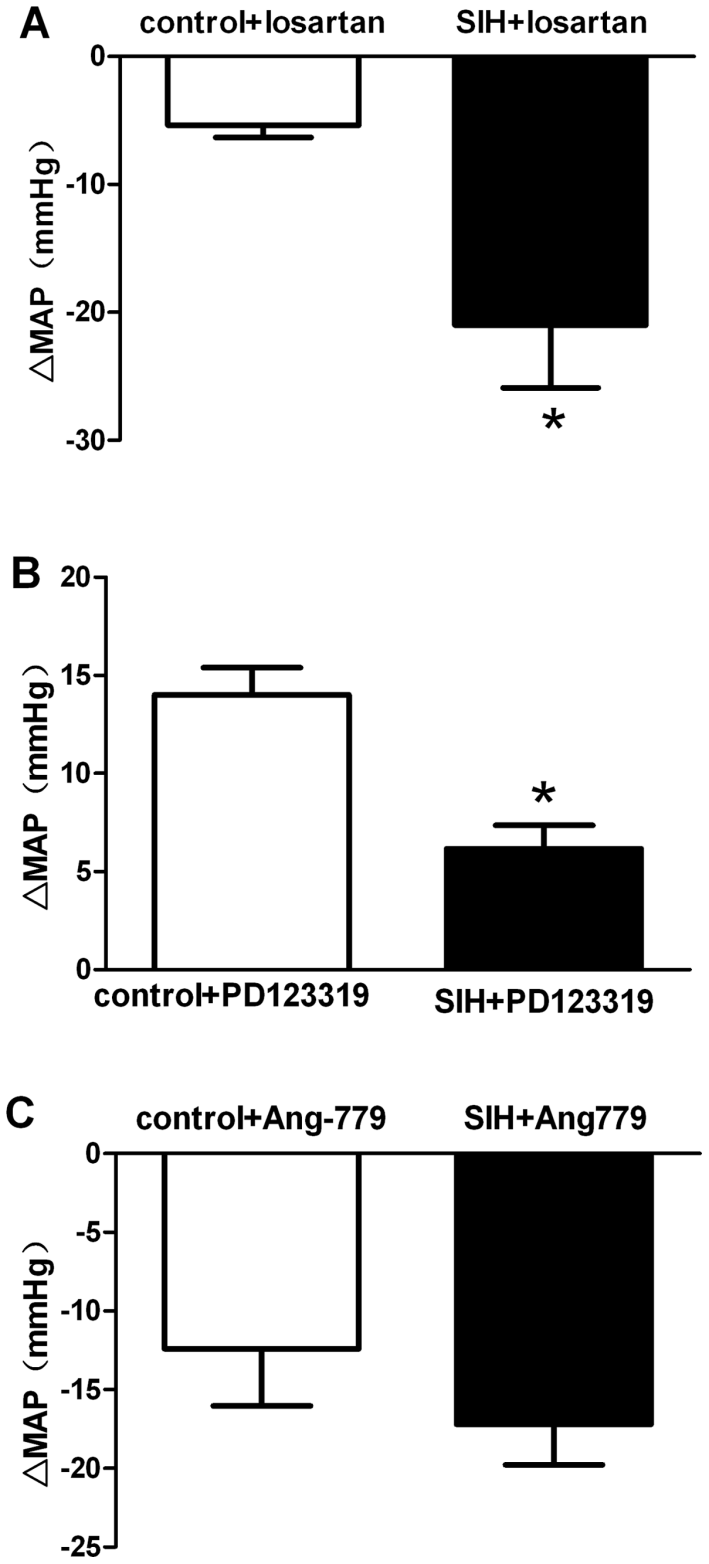
Effects of microinjection of losartan, PD123319 and Ang779 into the RVLM onΔMAP. *P<0.05, compared with the normotensive control group (n = 8 in each group).

To assess receptor activation under the baseline condition, AT_1_R antagonist losartan (1 nmol, Sigma, USA), AT_2_R antagonist PD123319 (1 nmol, Sigma, USA), or Mas receptor antagonist Ang779 (100 pmol, Sigma, USA) were microinjected into the RVLM. BP decreased in response to losartan in SIH but not in control rats (Fig. 4A, *P* *<*0.05), while BP increased in response to PD123319 in control rats but not in SIH rats (Fig. 4B, *P* *<*0.05). However, microinjection of Ang779 caused similar decreases in MAP in both SIH and control groups (Fig. 4C). Furthermore, losartan, PD123319 and Ang779 produced variable changes in HR. ΔHR was the same in SIH and control rats.

**Figure 5:**
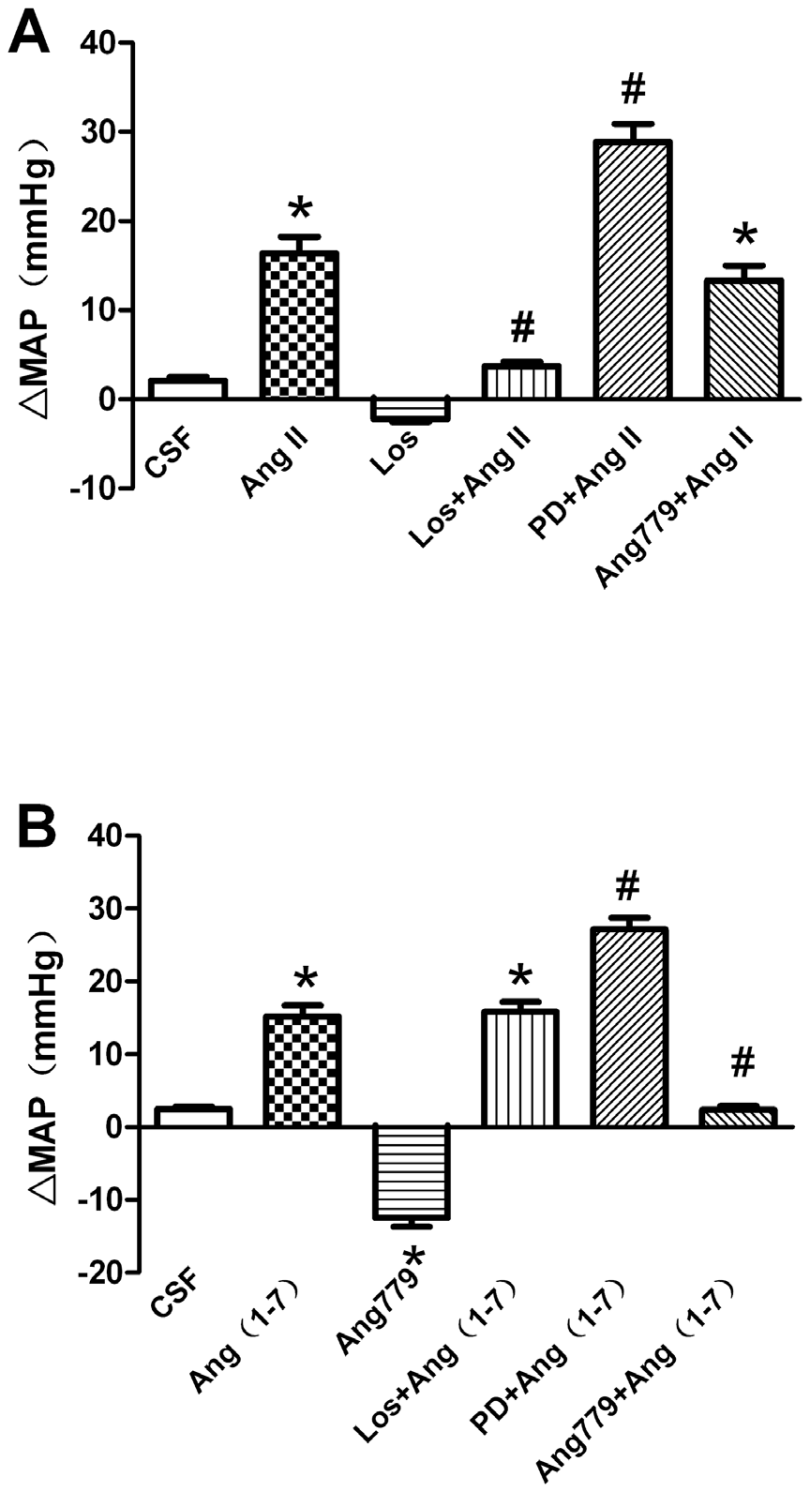
Effects of microinjection of Ang II, Ang-(1–7) alone or combined with their antagonists into the RVLM on ΔMAP. Los, losartan; PD, PD123319. *P<0.05, compared with artificial cerebrospinal fluid (CSF), *^#^P<*0.05, compared with Ang II or Ang-(1–7) alone (n = 6 for each group).

Pretreatment with losartant (1 nmol), but not PD123319 (1 nmol) or Ang779 (100 pmol), abolished the effect of Ang II (Fig. 5A), whereas Ang 779, but not the losartan or PD123319, eliminated the effect of Ang-(1–7) (Fig. 5B).

### 3. Molecular expressions

To determine the molecular mechanism, we assessed endogenous productions of Ang II and Ang-(1–7), and their receptor (AT_1_, AT_2_ and Mas) expressions, respectively in the RVLM of SIH and control rats. Since production of Ang II and Ang-(1–7) is determined by ACE and ACE2, we measured mRNA and protein expression.

**Figure 6:**
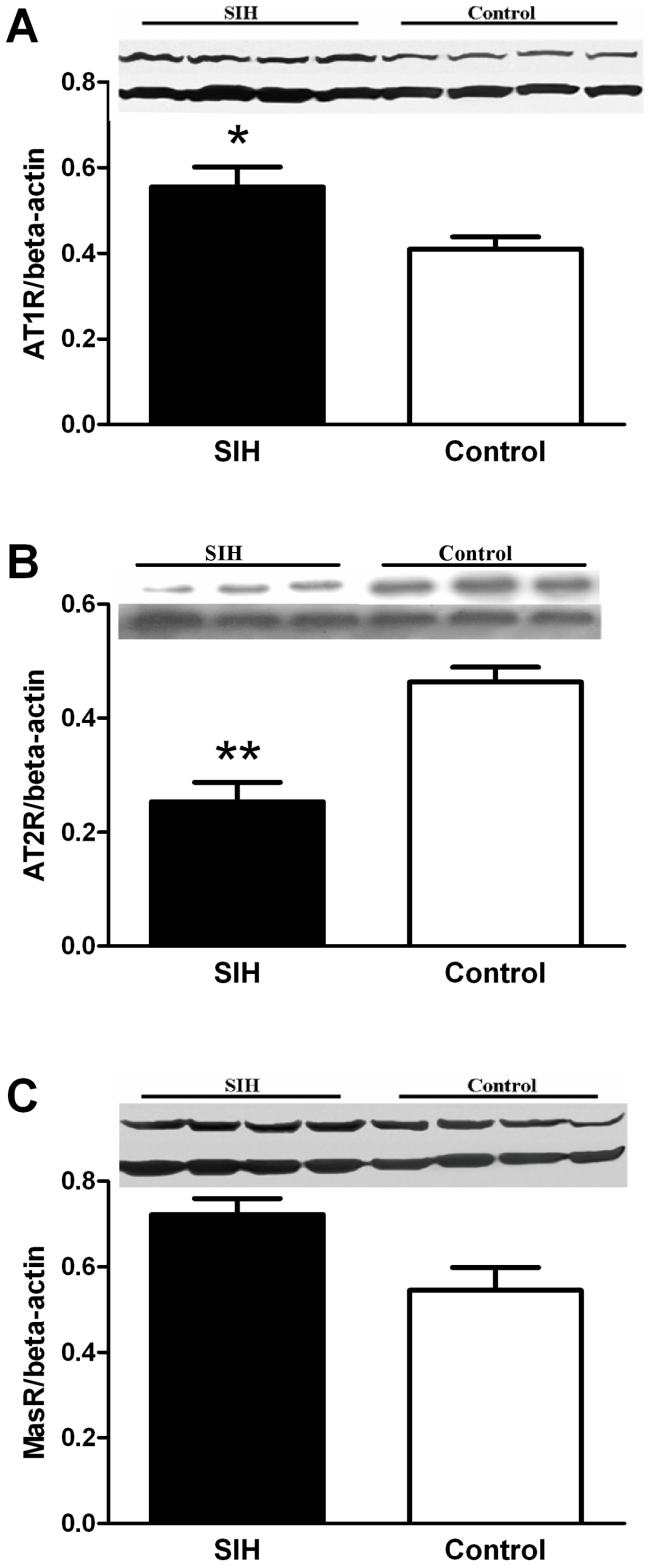
Expression levels of AT_1_, AT_2_ and Mas receptors in the RVLM. Data were normalized to β-actin. *P<0.05, compared with the normotensive control group (n = 3∼4 in each group).

**Figure 7:**
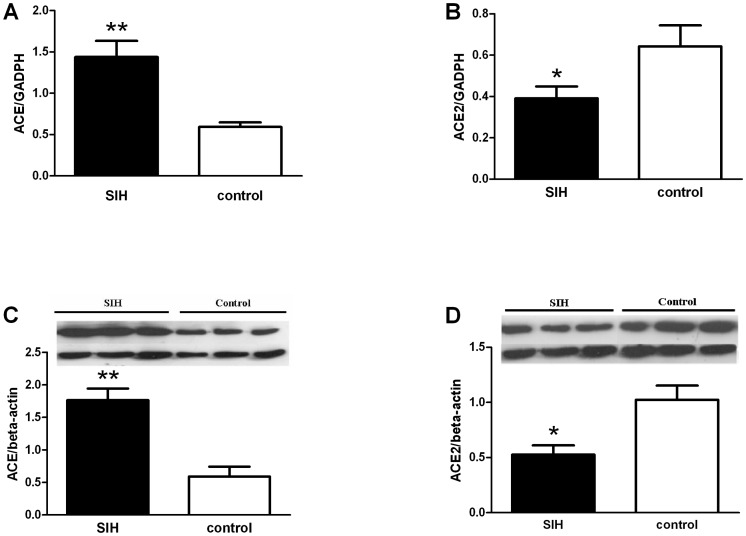
ACE, ACE2 mRNA and protein expressions in the RVLM. Data were normalized to GAPDH or β-actin. *P<0.05, compared with the normotensive control group (n = 3∼5 in each group).

Protein expression was detected by Western blot. AT_1_R expression was significantly greater and AT_2_R expression was significantly lower in the SIH group than the control group (Fig. 6A, B). However, Mas receptor expression was the same in both groups (Fig. 6C). ACE and ACE2 mRNA expression in the RVLM was measured by real-time PCR. Relative ACE mRNA and protein expression were significantly higher (*P<*0.01) (n = 5, Fig. 7A), (n = 3, Fig. 7C), whereas ACE2 mRNA and protein expression were significantly lower (*P<*0.05) (n = 5, Fig. 7B) (n = 3, Fig. 7D) in the SIH than the control group.

### 4. Effects of RAS on releases of amino acids

**Figure 8:**
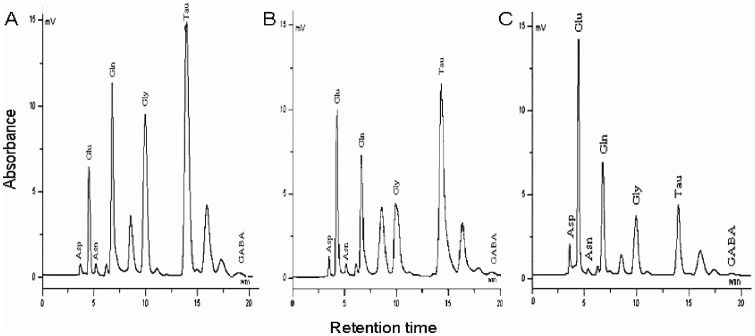
Chromatographic plots of amino acids in the RVLM following microinjection. ACSF in normal rats (A), Ang II in normal rat (B) or Ang II in SIH rat (C). The abscissa is retention time (min). The ordinate is absorbance. Asp, aspartate; Glu, Glutamate; Asn, asparagine; Gln, Glutamine; Gly, Glycine; Tau, taurine; GABA, γ-aminobutyric acid.

To establish that SIH is caused by alteration of RAS, which subsequently alters amino acid release leading to hemodynamic changes, we microinjected Ang II or Ang-(1–7) (and their antagonists) into the RVLM, and assessed amino acid releases in SIH and control rats. The amino acids examined in the PVLM included excitatory [glutamate (Glu) and aspartate (Asp)] as well as inhibitory [taurine (Tau), γ-aminobutyric acid (GABA) and glycine (Gly)] amino acids. The baseline release of excitatory amino acid neurotransmitters (Glu and Asp) increased but inhibitory amino acids (Tau and GABA) decreased in the SIH rats. Microinjection of Ang II (100 pmol) or Ang-(1–7) (100 pmol) increased Glu and decreased Tau and GABA releases (Table 2, 3; Fig. 8 B). However, Ang II effects were significantly greater in the SIH than the control group (*P<*0.05) (Fig. 8C, Table 2). Losartan significantly decreased Glu and increased Tau and GABA releases in SIH but not in control rats (Table 2). By contrast, microinjection of Ang-(1–7) or Ang779 caused comparable amino acid releases in the two groups (Table 3). No significant changes in Asp or Gly release were observed after microinjection of RAS components.

**Table 2:** Effects of microinjection of Ang II or losartan into the RVLM on local release of amino acids.

AA	Time	ACSF	AngII	Losartan	SIH+ACSF	SIH+AngII	SIH+losartan
Asp	baseline	5.5±0.5	5.7±0.7	6.1±0.8	8.3±0.6^#^	8.4±0.9^#^	8.5±0.8^#^
	1^st^ 10min	5.6±0.7	7.2±1.3	5.1±0.6	8.6±0.8^#^	10.5±1.5^#^	7.4±0.5^#^
	2^nd^ 10min	5.7±0.6	6.1±0.7	6.2±0.7	8.5±0.7^#^	9.6±0.9^#^	7.9±0.5^#^
	3^rd^ 10min	6.0±0.7	5.8±0.6	6.5±0.7	8.2±0.5^#^	8.7±0.8^#^	8.3±0.6^#^
Glu	baseline	21±1.8	20±1.8	21±2.2	34±2.9^#^	30±2.3^#^	31±2.4^#^
	1^st^ 10min	22±2.2	33±2.3* ^Δ^	17±2.7	38±3.1^#^	57±3.5* ^Δ#^	20±2.8* ^Δ^
	2^nd^ 10min	20±2.6	25±2.9	19±3.2	36±3.3^#^	47±4.8* ^Δ#^	28±2.9* ^#^
	3^rd^ 10min	23±2.3	22±2.4	22±1.9	34±2.8^#^	33±2.9^#^	30±2.6^#^
Gly	baseline	32±2.7	30±2.2	31±3.9	29±2.8	26±2.4	25±4.2
	1^st^ 10min	37±3.2	26±2.5	36±3.8	28±2.5	22±2.1	30±5.1
	2^nd^ 10min	34±4.9	27±3.8	34±4.3	25±2.2	26±4.9	32±4.3
	3^rd^ 10min	35±5.1	29±3.5	38±5.5	27±2.3	25±3.6	33±4.6
Tau	baseline	95±7.3	98±7.4	93±8.1	75±5.9^#^	75±5.2^#^	74±6.5^#^
	1^st^ 10min	96±7.4	81±7.2* ^Δ^	101±12	79±6.2^#^	41±4.5* ^Δ#^	95±8.4* ^Δ^
	2^nd^ 10min	93±7.3	87±6.9	97±9.3	78±6.1^#^	55±5.7* ^Δ#^	77±6.9^#^
	3^rd^ 10min	96±6.9	91±7.5	95±6.6	76±5.6^#^	70±5.2^#^	75±6.1^#^
GABA	baseline	5.1±0.3	5.2±0.4	5.2±0.6	3.6±0.4^#^	3.8±0.4^#^	3.6±0.3^#^
	1^st^ 10min	5.3±0.6	3.6±0.4* ^Δ^	6.0±0.7	3.4±.03^#^	1.2±0.2* ^Δ#^	5.1±0.5* ^Δ^
	2^nd^ 10min	5.4±0.3	4.7±0.5	5.8±0.4	3.4±0.4^#^	2.1±0.3*Δ^#^	4.0±0.3^#^
	3^rd^ 10min	5.3±0.4	5.1±0.4	5.3±0.5	3.7±0.5^#^	3.6±0.4^#^	3.7±0.4^#^

* *P*<0.05 compared with the ACSF group at the same time point; ^Δ^ *P*<0.05 compared with baseline (10 min prior to microinjection); ^#^ *P*<0.05 compared with the normotensive rats at the same time point. Amino acids (AA) were measured at baseline, 1^st^, 2^nd^, 3^rd^ 10 min after administration of ACSF, Ang II or losartan, respectively (n = 8 in each group). Asp, aspartate; Glu, Glutamate; Gly, Glycine; Tau, taurine; GABA, γ-aminobutyric acid.

**Table 3:** Effects of microinjection of Ang-(1–7) or Ang779 into the RVLM on local release of amino acids.

AA	Time	ACSF	Ang-(1–7)	Ang779	SIH+ACSF	SIH+Ang-(1–7)	SIH+Ang779
Asp	baseline	5.5±0.5	5.9±0.7	5.8±0.7	8.3±0.6^#^	8.2±0.8^#^	8.5±0.8^#^
	1^st^ 10min	5.6±0.7	7.0±1.5	5.4±0.6	8.6±0.8^#^	10.6±1.9^#^	7.5±0.9^#^
	2^nd^ 10min	5.7±0.6	6.4±0.8	6.0±0.5	8.5±0.7^#^	9.6±0.9^#^	7.1±0.5^#^
	3^rd^ 10min	6.0±0.7	6.1±0.6	6.1±0.6	8.2±0.5^#^	8.4±0.7^#^	8.2±0.6^#^
Glu	baseline	21±1.8	21±2.2	23±2.5	34±2.9^#^	32±3.1^#^	34±2.6^#^
	1^st^ 10min	22±2.2	35±3.1* ^Δ^	15±1.6* ^Δ^	38±3.1^#^	49±4.6* ^Δ#^	25±2.3* ^Δ#^
	2^nd^ 10min	20±2.6	28±2.5	21±2.2	36±3.3^#^	38±4.3^#^	31±3.0^#^
	3^rd^ 10min	23±2.3	24±2.9	24±2.1	34±2.8^#^	35±4.6^#^	32±2.7^#^
Gly	baseline	32±2.7	33±4.6	33±3.4	29±2.8	29±3.1	27±3.2
	1^st^ 10min	37±3.2	28±3.6	35±3.2	28±2.5	26±5.1	31±3.1
	2^nd^ 10min	34±4.9	31±3.4	32±4.3	25±2.2	27±4.5	30±3.3
	3^rd^ 10min	35±5.1	32±3.5	37±3.5	27±2.3	28±3.4	35±3.6
Tau	baseline	95±7.3	97±8.1	91±7.9	75±5.9^#^	77±6.5^#^	76±5.8^#^
	1^st^ 10min	96±7.4	80±7.4* ^Δ^	118±11* ^Δ^	79±6.2^#^	58±6.3* ^Δ#^	105±7.4* ^Δ^
	2^nd^ 10min	93±7.3	87±6.9	99±9.8	78±6.1^#^	69±7.1* ^#^	83±8.1
	3^rd^ 10min	96±6.9	95±6.8	94±6.9	76±5.6^#^	75±5.5^#^	77±6.9^#^
GABA	baseline	5.1±0.3	5.2±0.3	5.0±0.5	3.6±0.4^#^	3.6±0.6^#^	3.5±0.3^#^
	1^st^ 10min	5.3±0.6	3.8±0.4* ^Δ^	6.7±0.5* ^Δ^	3.4±.03^#^	2.2±0.4* ^Δ#^	5.4±0.5* ^Δ#^
	2^nd^ 10min	5.4±0.3	4.8±0.6	5.5±0.4	3.4±0.4^#^	3.3±0.5^#^	4.5±0.4^#^
	3^rd^ 10min	5.3±0.4	5.2±0.5	5.2±0.4	3.7±0.5^#^	3.5±0.4^#^	3.6±0.3^#^

* *P*<0.05 compared with the ACSF group at the same time point; ^Δ^ *P*<0.05 compared with baseline (10 min prior to microinjection); ^#^ *P*<0.05 compared with the normotensive rats at the same time point. Amino acids (AA) were measured at baseline, 1^st^, 2^nd^, 3^rd^ 10 min after administration of ACSF, Ang-(1–7) or Ang779, respectively (n = 8 in each group). Asp, aspartate; Glu, Glutamate; Gly, Glycine; Tau, taurine; GABA, γ-aminobutyric acid.

## Discussion

Hypertension affects approximately 1 billion individuals worldwide [25]. Chronic and excessive psychosocial stress can contribute to the development of hypertension. So it is very important to investigate the mechanism of SIH. SBP and AT_1_R protein expression in the RVLM increased in a time-dependent manner. ACE protein expression in the RVLM increased on the 15^th^ day. The SIH rat is a well established hypertensive model from our present and previous experiments [20], [21].

The RVLM is a cardiovascular integration center, which receives central and peripheral excitatory as well as inhibitory inputs to regulate hemodynamics [26]. The RAS plays an important role in this regulation and development of hypertension. Recently, two pathways, Ang II and Ang-(1–7), were identified in the RAS. These two pathways may interact synergistically or antagonistically [2], [10]. Microinjection of Ang II into the RVLM increased BP and sympathetic activity [23]. The pressor responses were usually greater in the spontaneously hypertensive rats (SHR) and Dahl salt-sensitive rats than in the Wistar-Kyoto (WKY) rats or Dahl salt-resistant rats [27], [28]. Endogenous Ang-(1–7) in the RVLM was also found to be important in the development of hypertension in the SHR and renovascular hypertensive rats [29], [30]. It is likely that both Ang II and Ang-(1–7) pathways participate in development of hypertension. However, it is not known about the relative importance of the two pathways and their effects on release of amino acid neurotransmitters in the SIH rats. The present studies investigated this issue and demonstrated that upregulation of Ang II by AT_1_R, not Ang-(1–7) pathway in the RVLM caused hypertension in SIH by increasing excitatory and suppressing inhibitory amino acid release. Our conclusion was based on the following analysis.

First, we showed that Ang II pathway, not Ang-(1–7) pathway was activated in SIH rats. This is demonstrated by the observation that Ang II microinjected into the RVLM increased BP, and the pressor effects were greater in SIH than in control rats. Furthermore, losartan decreased BP in SIH but not in control rats, while PD123319 increased BP in control but not in SIH rats. The results suggest that Ang II pathway was tonically activated under basal conditions in the SIH rat, while AT_2_R was tonically suppressed in the normotensive rat. Indeed, our results parallel with other reports that administration of AT_1_R antagonist (valsartan) into the RVLM could attenuate BP in the SHR but not in control WKY rats [31]. By contrast, microinjection of the Ang-(1–7) and its antagonist Ang779 into the RVLM elicited similar increases and decreases in BP in both SIH and normal rats, indicating that Ang-(1–7) pathway was not altered substantially during the development of SIH. The results of co-infused antagonists with their respective agonist experiments demonstrated that the pressor effect of Ang II in the RVLM was not mediated by AT_2_ or Mas receptors but by AT_1_ receptor. However, the pressor effect of Ang-(1–7) in the RVLM was not mediated by AT_1_ or AT_2_ receptor but by Mas receptor. These are similar to the other reports [32].

**Figure 9:**
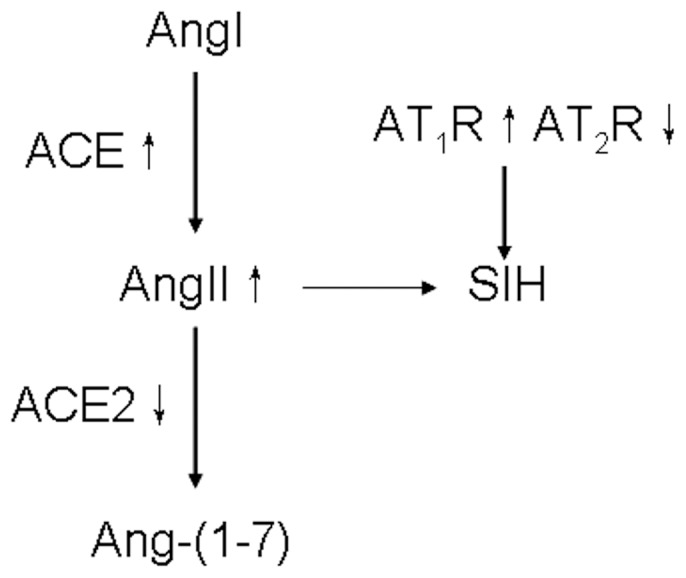
Schematic diagram showing the relationship between the renin-angiotensin system and stress-induced hypertension. ACE upregulation and/or ACE2 downregulation result in Ang II↑ and/or Ang-(1–7)↓. Ang, Angiotensin; ACE, angiotensin-converting enzyme; ACE2, angiotensin-converting enzyme homolog; AT_1_R, Ang II type 1 receptor; AT_2_R, Ang II type 2 receptor; SIH, stress-induced hypertension

Second, we showed that Ang II production was upregulated in SIH rats. We found that ACE mRNA and protein expression level were higher and ACE2 mRNA and protein expression level were lower in SIH than in normal rats. Such a molecular expression pattern in SIH rats could lead to an increased Ang II and decreased Ang-(1–7) production (Fig. 9). In addition, AT_1_R expression increased as AT_2_R expression decreased with no change in Mas receptor expression in SIH rats compared with normal rats. Thus, increased Ang II production and AT_1_R explain the increase in BP and a higher responsiveness to exogenous Ang II in SIH rats. Indeed, our molecular expression results correlate well with BP responses to microinjection of different components of RAS. Our data also agree with reports by other investigators. For example, AT_1_R in the RVLM was higher in the SHR than in WKY rats [16]. In the SHR and salt-sensitive Sabra hypertensive rats, ACE2 expression in the kidneys were markedly reduced [33]. Overexpression of brain-targeted ACE2 in the subfornical organ prevented pressor and drinking responses elicited by intracerebroventricular infusion Ang II [34], [35]. Yamazato et al. further demonstrated that ACE2 level in the RVLM decreased as hypertension develops in the SHR and increased BP can be reversed by overexpression of ACE2 with lentivirus [36].

Third, our data support that upregulation of Ang II pathway produces hypertension via an increase in excitatory and a decrease in inhibitory amino acid release. Many neurotransmitters (such as amino acids, Ang II, acetylcholine, and catecholamine) coexist in the RLVM. Excitatory amino acids (Glu and Asp) induce a pressor response [37], whereas inhibitory amino acids (Gly, Tau, and GABA) cause a depressor response [38]. Our previous studies found that both Ang II and Ang-(1–7) increased Glu release and BP in the normal rats [14], [15]. In the present study, the baseline releases of excitatory amino acids (Asp and Glu) were higher and inhibitory amino acids (Tau and GABA) were lower in the SIH than in the control group. We found release patterns of amino acids correlated well with pressor effects. For example, Ang II induced more changes in amino acids and BP in the SIH than in the control group. Losartan produced no change in release of amino acids and BP in normal rats. However, it decreased Glu and increased Tau and GABA releases, along with a decrease in BP in SIH rats. Since in the RVLM most glutamatergic and GABAergic neurons are co-labeled with AT_1_R and AT_1_R is located on the cell membrane and nerve processes, presynaptic mechanisms of AT_1_R may operate in the RVLM [13]. Therefore, Ang II may act on AT_1_R on the glutamatergic and GABAergic neurons to modulate amino acid release and BP.

In summary, in the present studies we investigated two RAS pathways [Ang II and Ang-(1–7)] in the RVLM in the development of SIH. We found SIH rats were more responsive to microinjection of Ang II and AT_1_R antagonist [but not Ang-(1–7) and Mas receptor antagonist], expressed more AT_1_R (but not Mas receptor), ACE and less AT_2_R, ACE2, and caused increased release of excitatory and decreased release of inhibitory amino acids in response to Ang II. Thus, we conclude that activation of Ang II pathway (increased Ang II production and AT_1_R) contributes significantly to hypertension in SIH by increasing excitatory and suppressing inhibitory amino acid release.
